# Treatment Strategies for Cancer Patients in Post-Peak of the Novel Coronavirus Disease (COVID-19) Period in China

**DOI:** 10.3389/fonc.2020.00925

**Published:** 2020-05-28

**Authors:** Binliang Liu, Hongnan Mo, Fei Ma

**Affiliations:** Department of Medical Oncology, National Cancer Center/National Clinical Research Center for Cancer/Cancer Hospital, Chinese Academy of Medical Sciences and Peking Union Medical College, Beijing, China

**Keywords:** treatment strategies, COVID-19, post-peak, China, cancer

## Introduction

Since its outbreak in China in December 2019, the novel coronavirus disease (COVID-19) has spread rapidly worldwide (1). The World Health Organization (WHO) declared the COVID-19 outbreak a pandemic on March 11, 2020. By May 8, 2020, the virus had caused 3,759,967 cases and 259,474 deaths (2). Cancer patients are at a higher risk of getting COVID-19. When cancer patients are infected, cancer treatments need to be rearranged. Since they are also at risk of serious illness from COVID-19, their progression of symptoms could be faster (3–5).

In order to reduce and avoid cross-infection of COVID-19, many hospitals have taken measures to reduce the number of afebrile patients. One of the measures that have affected the vast majority of cancer patients is delaying or suspending cancer treatment, which is dangerous for such patients.

To ensure the cancer patients get essential treatments, as early as February 17, 2020, the National Health Commission issued a notice that emphasizes the necessity of providing daily medical services and maintaining reasonable medical functioning while fighting the epidemic. It also mentioned that rigidly ceasing daily medical services is neither wise nor advisable. Due to the excellent efforts of isolation, quarantine, social distancing, and community containment in the last 3 months (1, 6), the number of new cases of COVID-19 in Hubei Province was decreased to zero for the first time on March 18. Since then, new cases reported in China mainly came from abroad. The situation of China's epidemic prevention and control has entered a new period, a post-peak period.

Now, it is urgent to resume the routine treatment of cancer patients. Under the background of occupied medical resources and high risks of infection, we should strive to “manage epidemics and anti-cancer at the same time.” Here, based on our own experience, we summarize how to deal with the rapidly growing needs of cancer patients in the post-peak period of COVID-19 in this Opinion.

## Surgical Treatment

Considering that, during the epidemic, it is easy when doing operations to cause widespread infections among doctors, nurses, anesthesiologists, and patients in the operating room, only emergency or essential surgeries can be carried out. Most non-essential or elective surgeries are postponed during the outbreak period (7, 8). At present, semi-elective and elective surgeries are gradually coming back to normal. However, patients who need surgery need to be hospitalized and observed for 7 days before surgery. To perform or not to perform an operation requires careful consideration of the patient's condition, the risk of the proposed operation, the stock of blood banks, and the storage of protective materials. It is recommended to restore the performance of outpatient surgeries involving the superficial organs (skin, thyroid, and breast) first.

## Radiotherapy

During the epidemic period, most radiotherapies have been stopped. The unique challenges of radiation therapy are that it cannot be interrupted and cannot be performed at home.

With the improvement of the epidemic situation in China, radiotherapy needs to be started for patients when their treatments cannot be exempted or delayed. The recommended radiotherapy plan is hypofractionated radiotherapy, which could reduce the frequency of hospital visits. However, at the same time, attention must be paid to such a plan to minimize the chances of lung injury.

## Systematic Therapy

During the epidemic period, the treatment strategy for cancer patients tends to be “moderate.” Oral drug treatment [including oral chemotherapy, endocrine therapy, and metronomic treatment (9)] and targeted treatments are the mainstream. At present, intravenous treatment is just starting up again, and outpatients could be given priority to reserve a place for chemotherapy. At the same time, we are beginning to treat some inpatients, and all inpatients need to be carefully screened before admission. The hospital bed density needs to be kept at a low level for now, but the number of inpatients could be increased gradually in the future.

Before treatment, it is highly recommended to complete a pre-screening for COVID-19 for all patients, including checks of body temperature, blood pressure, and oxygen saturation. Patients also need to fill up a questionnaire inquiring about the history of living in the epidemic area, the contact history with confirmed/suspected cases of COVID-19, and the occurrence of COVID-19-like symptoms such as fever, cough, sputum, and diarrhea. CT screening and a coronavirus nucleic acid test should be carried out if the patient shows any symptoms or has COVID-19 exposure risk.

After treatment, the monitoring and management of adverse drug reactions need to be more thoughtful. The frequency of follow-up calls should be increased, especially when a long-term prescription policy is still in place during the post-peak period. Treatments with side-effects showing pneumonia-like symptoms or triggering pneumonia should be avoided, such as PD-1/PD-L1 and Everolimus.

At present, the situation of China's domestic epidemic prevention and control continues to improve. The activities of manufacturing industries and people's daily lives have quickly been restored. The successful experience of fighting COVID-19 in China shows that COVID-19 is controllable under proper management. We hope that the Chinese experience can provide valuable information to help oncologists and cancer patients around the world in the peak and post-peak period of COVID-19. What the international community needs are firm confidence, concerted efforts, and a united response. We will win the fight against COVID-19.

## Discussion

We share our experience to provide a reference for other hospitals that need to restore the treatment of cancer patients in the post-peak period of COVID-19. However, approaches to restarting the diagnosis and treatment of cancer patients should be tailored according to the actual situation of different countries/regions/hospitals, since the severity of COVID-19 and its impact on healthcare systems are not the same. The management guidelines or suggestions for cancer patients from other countries during the epidemic period or post-peak period are also of constructive significance to us (8, 10, 11). Measures that are beneficial to anti-tumor treatment and are not against the principle of epidemic control are all worth discussing.

In order to restart the treatment of cancer patients in a more reasonable and timely manner, local authorities should also issue and update guidelines and schedules for opening up the hospitals step by step, which is an excellent aid to restoring medical activity to normal.

## Author Contributions

BL and FM: conception and design. BL: manuscript drafting. HM and FM: manuscript revision. All authors: manuscript reviewing.

## Conflict of Interest

The authors declare that the research was conducted in the absence of any commercial or financial relationships that could be construed as a potential conflict of interest.
